# Two Infants With Beta-Ketothiolase Deficiency Identified by Newborn Screening in China

**DOI:** 10.3389/fgene.2019.00451

**Published:** 2019-05-15

**Authors:** Yuqi Yang, Shu hong Jiang, Shuang Liu, Xiao ya Han, Ying Wang, Lei lei Wang, Bin Yu

**Affiliations:** ^1^Changzhou Maternity and Child Health Care Hospital, Nanjing Medical University, Changzhou, China; ^2^Lianyungang Maternal and Child Health Hospital, Yangzhou University, Lianyungang, China

**Keywords:** newborn screening, inborn errors of metabolism, tandem mass spectrometry, beta-ketothiolase deficiency, next-generation sequencing, *ACAT1*

## Abstract

Beta-ketothiolase deficiency (BKTD) is an autosomal recessive disease caused by a defect of mitochondrial acetoacetyl-CoA thiolase. Beginning in 2014, we carried out newborn screening by tandem mass spectrometry (MS/MS) followed by next-generation sequencing (NGS) and identified two infants with BKTD among 203,750 newborns born in Jiangsu Province, China. Both infants showed the characteristic chemical abnormalities of BKTD. We used NGS to confirm variants in the *ACAT1*. Patient 1 had the compound heterozygous variants c.721dupA and c.928G > C. Patient 2 had compound heterozygosity for the c.238+1G > A and c.1163G > T variants. c.721dupA, c.928G > C and c.1163G > T were suspected to be likely pathogenic, whereas c.238+1G > A was determined to be pathogenic. None of the four variants have been reported in the literature. Patient 1 presented with onset of metabolic acidosis and neonatal hypoglycemia 8 days after birth, whereas patient 2 was detected through neonatal disease screening but had no clinical manifestations. These findings contribute to our understanding of the clinical characteristics and genetic basis of BKTD.

## Introduction

Beta-ketothiolase deficiency (MIM 203750) occurs due to a mitochondrial acetoacetyl-CoA thiolase (T2; EC2.3.1.9) defect caused by mutation of *ACAT1* (NG_009888, NM_000019.3), leading to the abnormal metabolism of isoleucine and ketone. The incidence of BKTD is very low. Since its first description in 1971, about 135 cases have been reported (Fukao et al., 2019). Most patients have no obvious clinical manifestations in the early postnatal period. The age of onset is usually 6 to 18 months (Abdelkreem et al., 2016; Nguyen et al., 2017), and patients grow well before the first attack. Timely intervention and treatment before the first episode can enable full recovery or prolong the period until symptoms develop. The clinical manifestations of BKTD are non-specific and variable, with the most common clinical feature being acute ketoacidosis (Sass, 2012). Because most patients with BKTD do not exhibit significant clinical symptoms, newborn screening by tandem mass spectrometry (MS/MS) may be the only approach for early detection.

Since 2014, we have performed newborn screening for BKTD by MS/MS followed by next-generation sequencing (NGS) for genetic diagnosis. The study design and protocol were reviewed and approved by the Ethics Committee of the Changzhou Maternity and Child Health Care Hospital, affiliated with Nanjing Medical University (No. 201502). Written informed consent was obtained from all of the infants’ parents before newborn screening. Over 5 years, we screened 203,750 infants and diagnosed two cases of BKTD, as described herein.

## Case Presentation

### Patient 1

A woman gave birth to a full-term infant of 3800 g by cesarean section at a gestational age of 38^+6^ weeks. The boy was 49 cm long, with a head and chest circumference of 33 and 32 cm, respectively. Apgar scores in the first and the fifth minutes were both 9. The mother and her spouse had no family history of BKTD. Their first child was a phenotypically normal girl. This boy was admitted to the hospital 8 days after birth with rapid breathing, poor reaction, and spitting for 2 days. A hospital physical examination found a lack of alertness, paleness, and shortness of breath but no cyanosis. Pulmonary auscultation revealed crackles in both lung fields. He also had reduced muscle tone and swelling of the right heel. In auxiliary examinations, the results of routine blood examination, urine tests (including urine ketone body test), and tests for hepatic and renal function were normal. Values of biochemical parameters in the cerebrospinal fluid were also normal. Cerebrospinal fluid bacterial culture was negative. A blood gas analysis indicated metabolic acidosis and neonatal hypoglycemia with an arterial pH of 6.783, PCO_2_ of 16.3 mmHg, PO_2_ of 315 mmHg, lactic acid of 2.5 mm/L, BE of –30.4 mmol/L, and glucose level of 2.1 mmol/L. A chest radiograph showed increased lung markings.

Written informed consent was obtained from the patient’s parents before screening for participation in and publication of this case report. The baby received newborn screening by MS/MS 3 days after birth. The concentrations of malonylcarnitine+3-hydroxybutyrylcarnitine (C3DC+C4OH), methylmalonylcarnitine+3-hydroxyisovalerylcarnitine (C4DC+ C5OH), and tiglylcarnitine (C5:1) exceeded the cut-off values (Table 1). Urinary organic acid analysis by gas chromatography-mass spectrometry (GC-MS) showed increased levels of 2-methyl-3-hydroxybutyric acid and methylcrotonylglycine at 44.14 (reference range, 0.0–0.3) and 9.8 (reference range, 0.0–0.1), respectively. The MS/MS results and urine organic acid levels were highly indicative of BKTD.

**TABLE 1:** Biochemical criteria and clinical manifestations.

Case	Age at diagnosis	Age of onset	Acyl carnitine in blood (μmol/L)			Acid metabolites in urine		Clinical manifestations				
			C3DC+C4DC	C4DC+C5OH	C5:1	2-Methyl-3-hydroxybutyricacid	Methylcrototonyl-glycine	Metabolic acidosis	Coma	Vomiting	Neurologic manifestations	Other
Case 1	<1 month	8 days	3.58	1.31	0.31	4.14	9.8	Present	Absent	Absent	Normal	Hypoglycemia
Case 2	<1 month	None	1.22	0.71	0.2	14.08	5.74	Absent	Absent	Absent	Normal	Absent

*Urinary organic acids are given as ratios, so there is no unit. Reference ranges: C3DC+C4DC (0.02–0.3); C4DC+C5OH (0.06–0.4); C5:1 (0–0.02); 2-methyl-3-hydroxybutyric acid (0.0–0.3); methylcrototonylglycine (0.0–0.1).*

**FIGURE 1:**
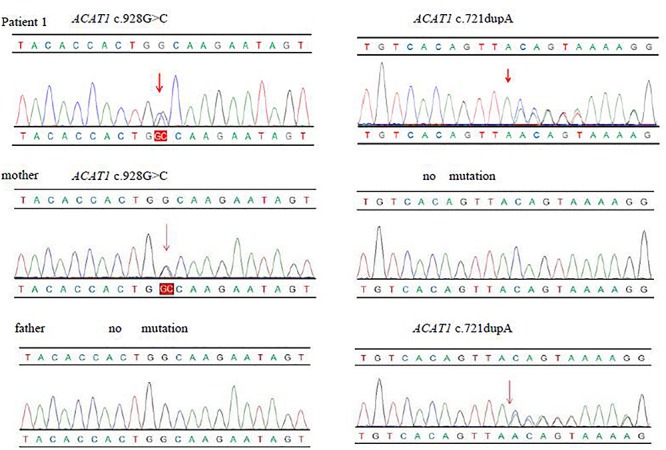
The variants of ACAT1 gene in patient and his family.

We used NGS to identify pathogenic mutations. The baby had the complex heterozygous variants c.721dupA (p.Thr241Asn fs*14), located in exon 7, and c.928G > C (p.Ala310Pro), located in exon 9 in the *ACAT1*. We also found the heterozygous mutations c.804del in *SL*C*25A20* and c.799C > T in *HMGCL*. We verified the mutations in *ACAT1* in the baby and his family members by Sanger sequencing. The c.721dupA variant was inherited from his father, and the c.928G > C variant was inherited from his mother. His sister only inherited c.721dupA from the father and had no clinical manifestations as an asymptomatic carrier (Figure 1). We used the Genome Analysis Toolkit^1^ to identify the mutation sites in the target sequence and Annovar^2^ to annotate all mutation sites within the public database. The influence of the mutations on protein function was predicted according to the frequency of the mutations in the normal population, the conservation of the mutated sequence, and the amino acid changes caused by the mutations as well as their locations in the protein structure. Finally, combined with the sample’s own situation, we inferred the pathogenicity of each mutation based on the ACMG standards and guidelines. We determined that the variants in patient 1 may affect the function in the *ACAT1* protein. c.721dupA and c.928G  >  C were identified as likely pathogenic (Table 2).

**TABLE 2:** Characteristics of disease-causing genetic variants.

			**Effect on amino**		
**Case**	**Mutation location**	**Nucleotide change**	**acid sequence**	**Clinical significance**	**Source of variation**
Case 1	chr11:108010933–108010934	c.721dupA	p.Thr241Asn fs*14	Likely pathogenic	Father
	chr11:108013265	c.928G > C	p.Ala310Pro	Likely pathogenic	Mother
Case 2	chr11:108004665	c.238+1G > A	/	Pathogenic	Not determined
	chr11:108017086	c.1163G > T	P.Gly388Val	Likely pathogenic	Not determined

*Transcript ID: NM_000019.3.*

At the time of diagnosis, we treated the initial clinical symptoms, such as neonatal pneumonia, neonatal hypoglycemia, and metabolic acidosis. Specific measures included oxygen inhalation therapy, correction of acidosis with sodium bicarbonate, relief of heart burden using furosemidum, infection control, and fluid replacement therapy. These treatments substantially relieved the child’s symptoms. After a definitive diagnosis of BKTD, the patient was administered enough glucose to reduce protein breakdown and carnitine to reduce excessive acid metabolites. After discharge, the patient was instructed to limit protein intake [1–2 g/(kg⋅d)] and avoid hunger and was administered oral l-carnitine [dosage of 50–100 mg/(kg⋅d)]. The patient was followed up every 3 months, and the dosage of l-carnitine was adjusted according to the levels of C3DC+C4OH, C4DC+C5OH, and C5:1. The boy is now 15 months old. No serious complications have occurred during the year of follow-up.

### Patient 2

Patient 2 was 50 cm in length at birth, with a head circumference of 33 cm and chest circumference of 32 cm. His Apgar scores at 1 and 5 min were both 9. Written informed consent was obtained from the patient’s parents before screening for participation in and publication of this case report. Based on newborn screening by MS/MS, C3DC+C4OH and C4DC+C5OH levels and the C5:1 ratio in the blood were elevated, similar to the results for patient 1. High levels of 2-methyl-3-hydroxybutyric acid were also detected in his urine by GC-MS (Table 1). We found two variants in the *ACAT1*, with mutations c.238+1G > A and c.1163G > T (P.Gly388Val) (Table 2). The c.238+1G > A mutation of *ACAT1* is a splice site mutation, altering the nucleotide at position 1 of the 5′ end of intron 2. According to ACMG guidelines, classical splice-site mutations disrupt gene functions, e.g., by an inability to transcribe or synthesize the corresponding products (Richards et al., 2015). c.1163G > T is a missense mutation predicted to be likely pathogenic. Unlike patient 1, patient 2 did not have any clinical manifestations. His parents refused further genetic analysis and follow-up visits, and thus additional clinical information on the child is lacking. We did not intervene medically, other than recommending close follow-up. The boy has not yet exhibited severe clinical manifestations.

## Discussion

It is generally believed that BKTD lacks specific clinical manifestations. Most affected children suffer from diseases caused by fasting, fever, infection, and other issues, which result in sudden ketoacidosis (Thummler et al., 2010; Hori et al., 2015). The age of first onset of ketoacidosis in most cases is 6 to 18 months. Fukao (Fukao et al., 2014) claimed that mitochondrial medium-chain 3-ketoacyl-CoA thiolase (T1) can partially compensate for the T2 deficiency. Ketogenic triggers, such as prolonged fasting, infection, and protein-rich foods, may disrupt this compensation and precipitate acute ketoacidosis. Frequent feeding, protective maternally acquired immunoglobulins, and the relatively low protein contents of breast and ordinary milk formulas reduce the risk of ketogenic triggers (Nguyen et al., 2017). Therefore, the sooner affected children receive intervention after birth, the better the control of the disease.

Some BKTD patients present with neurological symptoms before the onset of ketoacidosis (Buhas et al., 2013), whereas others exhibit other metabolic abnormalities, such as hypoglycemia, hyperglycemia, or high blood ammonia. In this study, patient 1 developed severe metabolic acidosis and hypoglycemia at 8 days after birth due to a pulmonary infection. The age of onset was earlier than the average reported in the literature. Neonatal onset of T2 deficiency is very rare. Fukao et al. (2019) reported only two cases with neonatal onset out of 135 patients with T2 deficiency. Acidosis, hypoglycemia, and unconsciousness occurred in one case during the first episode, similar to that observed in patient 1 in this study. Ketonuria is usually evident in patients with BKTD during acute ketoacidosis; however, patient 1 had no detectable ketonuria. This may occur if urinary testing was performed after correcting the metabolic derangements. Another possible cause may be the coexistence of another inherited defect in ketogenesis or beta–oxidation. However, NGS did not reveal biallelic variants in relevant genes. Only one *SLC25A20* variant (c.804del) was identified in patient 1; if an additional *SLC25A20* variant was detected, we might attribute the absent ketonuria in this patient to a coexistent carnitine-acylcarnitine translocase deficiency. Secondary carnitine deficiency that may suppress beta-oxidation might be another explanation (Aghamaleki et al., 2019). However, the coexistence of secondary carnitine deficiency in patient 1 is unlikely because he did not have low carnitine levels.

According to current reports, most children with BKTD exhibit abnormalities in urinary organic acid and blood acyl carnitine. Meanwhile, some studies have reported only subtle abnormalities, even during acute episodes (Fukao et al., 2012). The detection of enzyme activity in fibroblasts using “coupled assay” test cannot be used to distinguish T2 deficiency from 2-methyl-3-hydroxybutyryl-CoA dehydrogenase (MHBD) deficiency (Gibson et al., 1992). Some patients with residual enzyme activity may also be missed (Zhang et al., 2004). T2 and MHBD deficiencies may result in similar urinary organic acid abnormalities (Fukao et al., 2014). Therefore, BKTD cannot be diagnosed based only on urine test results or the detection of enzyme activity. Fortunately, there are other ways to identify T2 and MHBD deficiency. For example, 2-methylaceoacetate is only detected in T2 deficiency, and the potassium-dependent AA-CoA thiolase assay gives a normal result in cases of MHBD deficiency (Haapalainen et al., 2007). Unfortunately, the results of these tests were not available in our patients, and this represents a limitation of our study. Beyond that, genetic testing is another important way to distinguish T2 and MHBD deficiencies. This was exactly what our research uses.

Newborn screening is highly effective for the detection of diseases in children who lack clinical symptoms. In the 1990s, the categories of inherited metabolic diseases included in screening were expanded by the introduction of MS/MS (Frazier et al., 2006). Likewise, NGS enables the sequencing of hundreds of thousands to millions of DNA molecules simultaneously and can be used to detect variations in disease-causing genes simply and accurately. In China, the combination of MS/MS and NGS is considered to be a superior method for newborn screening. Beginning in 2014, we screened for diseases in this way and have successfully diagnosed and intervened in many metabolic diseases (Yang et al., 2019). In this study, MS/MS results suggested the possibility of BKTD, but abnormalities in blood acylcarnitine are not specific to BKTD. Therefore, we performed related auxiliary examinations based on clinical manifestations including urine gas chromatography, a blood biochemistry test, and blood glucose test. At the same time, we performed genetic testing and mutation site validation on the children and their families to confirm BKTD. Both patient 1 and patient 2 had compound heterozygous mutations of *ACAT1* (c.721dupA and c.928G > C in patient 1; c.238+1G > A and c.1163G > T in patient 2), none of which have been reported in the literature. Of course, normal newborn screening does not absolutely exclude BKTD.

The *ACAT1* is located on chromosome 11q22.3 to q23.1. It is 27 kb in length and is composed of 12 exons and 11 introns (Kano et al., 1991). Variations of *ACAT1* in patients are diverse, and genotypes are not related to clinical manifestations (Nguyen et al., 2017). In Asia, it has been reported that the mutation frequency of c.1124A > G in the Chinese population is relatively high, whereas c.266G > C is the mutation with the highest frequency in Vietnam (Fukao et al., 2010) and c.578T > G is the most common mutation in India (Abdelkreem et al., 2017). Since the pathogenicity of the four new mutations detected in this study was predicted by software (e.g., SIFT, PolyPhen2, PROVEAN, and Mutation Taster), their pathogenicity should be further confirmed by *in vitro* protein functional expression assays. Meanwhile, we are submitting the four novel *ACAT1* variants to the public database (ClinVar).

Previous reports have indicated that the incidence of BKTD is less than 1 per 1 million newborns. However, data from newborn screening from many countries has shown that the incidence is higher than previously estimated (Sarafoglou et al., 2011). Since 2014, our laboratory has screened more than 200,000 newborn babies and identified two cases, resulting in an incidence of BKTD of 1:101,875. China has not yet popularized MS/MS screening, and therefore reliable data on the incidence of BKTD among the Chinese population is lacking. However, an increasing number of newborn screening centers in China have begun performing this test (Han et al., 2007; Sun et al., 2011; Guo et al., 2018; Yang et al., 2018). We believe that future studies on the Chinese population will provide useful information and a clearer understanding of BKTD.

## Ethics Statement

The study design and protocol were reviewed and approved by the Ethics Committee of the Changzhou Maternity and Child Health Care Hospital, Nanjing Medical University (No. 201502). We obtained written informed consent for genomic analysis from the parents of the baby and the mother of the patient provided written informed consent for the publication of this case report.

## Author Contributions

BY and LW carried out the assays and participated in the study design. YY, SJ, SL, XH, and YW carried out clinical consultations, laboratory tests, and performed the statistical analysis. BY conceived the study, participated in its design and coordination and helped draft the manuscript.

## Conflict of Interest Statement

The authors declare that the research was conducted in the absence of any commercial or financial relationships that could be construed as a potential conflict of interest.

## Footnotes

1 https://software.broadinstitute.org/gatk/

2 http://annovar.openbioinformatics.org/en/latest/

## References

[B1] Abdelkreem E. Akella R. R. D. Dave U. Sane S. Otsuka H. Sasai H. (2017). Clinical and mutational characterizations of ten indian patients with beta-ketothiolase deficiency. *Jimd Rep.* 35 59–65. 10.1007/8904_2016_26 27928777 PMC5585108

[B2] Abdelkreem E. Otsuka H. Sasai H. Aoyama Y. Hori T. Abd El A. M. (2016). Beta-ketothiolase deficiency: resolving challenges in diagnosis. *J. Inborn Errors Metab. Screen.* 4 1–9. 10.1177/232640981663

[B3] Aghamaleki M. A. Sasai H. Abdelkreem E. Ago Y. Amiri S. S. Moslemi L. (2019). Beta-ketothiolase deficiency: a case with unusual presentation of non-ketotic hypoglycemic episodes due to coexistent probable secondary carnitine deficiency. *JIMD Rep.* 46 23–27. 10.1002/jmd2.12022 PMC6498828 31240151

[B4] Buhas D. Bernard G. Fukao T. Decarie J. C. Chouinard S. Mitchell G. A. (2013). A treatable new cause of chorea: beta-ketothiolase deficiency. *Mov. Disord.* 28 1054–1056. 10.1002/mds.25538 23818432

[B5] Frazier D. M. Millington D. S. McCandless S. E. Koeberl D. D. Weavil S. D. Chaing S. H. (2006). The tandem mass spectrometry newborn screening experience in North Carolina: 1997-2005. *J. Inherit. Metab. Dis.* 29 76–85. 10.1007/s10545-006-0228-9 16601872

[B6] Fukao T. Maruyama S. Ohura T. Hasegawa Y. Toyoshima M. Haapalainen A. M. (2012). Three japanese patients with beta-ketothiolase deficiency who share a mutation, c.431A > C (H144P) in ACAT1 : subtle abnormality in urinary organic acid analysis and blood acylcarnitine analysis using tandem mass spectrometry. *JIMD Rep.* 3 107–115. 10.1007/8904_2011_72 23430882 PMC3509868

[B7] Fukao T. Mitchell G. Sass J. O. Hori T. Orii K. Aoyama Y. (2014). Ketone body metabolism and its defects. *J. Inherit. Metab. Dis.* 37 541–551. 10.1007/s10545-014-9704-9 24706027

[B8] Fukao T. Nguyen H. T. Nguyen N. T. Vu D. C. Can N. T. Pham A. T. (2010). A common mutation, R208X, identified in vietnamese patients with mitochondrial acetoacetyl-CoA thiolase (T2) deficiency. *Mol. Genet. Metab.* 100 37–41. 10.1016/j.ymgme.2010.01.007 20156697

[B9] Fukao T. Sasai H. Aoyama Y. Otsuka H. Ago Y. Matsumoto H. (2019). Recent advances in understanding beta-ketothiolase (mitochondrial acetoacetyl-CoA thiolase, T2) deficiency. *J. Hum. Genet.* 64 99–111. 10.1038/s10038-018-0524-x 30393371

[B10] Gibson K. M. Lee C. F. Kamali V. Sovik O. (1992). A coupled assay detecting defects in fibroblast isoleucine degradation distal to enoyl-CoA hydratase: application to 3-oxothiolase deficiency. *Clin. Chim. Acta.* 205 127–135. 1355701 10.1016/s0009-8981(05)80007-5

[B11] Guo K. Zhou X. Chen X. Wu Y. Liu C. Kong Q. (2018). Expanded newborn screening for inborn errors of metabolism and genetic characteristics in a chinese Population. *Front. Genet.* 9:122. 10.3389/fgene.2018.00122 29731766 PMC5920142

[B12] Haapalainen A. M. Merilainen G. Pirila P. L. Kondo N. Fukao T. Wierenga R. K. (2007). Crystallographic and kinetic studies of human mitochondrial acetoacetyl-CoA thiolase: the importance of potassium and chloride ions for its structure and function. *Biochemistry* 46 4305–4321. 10.1021/bi6026192 17371050

[B13] Han L. S. Ye J. Qiu W. J. Gao X. L. Wang Y. Gu X. F. (2007). Selective screening for inborn errors of metabolism on clinical patients using tandem mass spectrometry in China: a four-year report. *J. Inherit. Metab. Dis.* 30 507–514. 10.1007/s10545-007-0543-9 17347912

[B14] Hori T. Yamaguchi S. Shinkaku H. Horikawa R. Shigematsu Y. Takayanagi M. (2015). Inborn errors of ketone body utilization. *Pediatr. Int.* 57 41–48. 10.1111/ped.12585 25559898

[B15] Kano M. Fukao T. Yamaguchi S. Orii T. Osumi T. Hashimoto T. (1991). Structure and expression of the human mitochondrial acetoacetyl-CoA thiolase-encoding gene. *Gene* 109 285–290. 1684944 10.1016/0378-1119(91)90623-j

[B16] Nguyen K. N. Abdelkreem E. Colombo R. Hasegawa Y. Can N. T. Bui T. P. (2017). Characterization and outcome of 41 patients with beta-ketothiolase deficiency: 10 years’ experience of a medical center in northern Vietnam. *J. Inherit. Metab. Dis.* 40 395–401. 10.1007/s10545-017-0026-6 28220263

[B17] Richards S. Aziz N. Bale S. Bick D. Das S. Gastier-Foster J. (2015). Standards and guidelines for the interpretation of sequence variants: a joint consensus recommendation of the american college of medical genetics and genomics and the association for molecular pathology. *Genet. Med.* 17 405–424. 10.1038/gim.2015.30 25741868 PMC4544753

[B18] Sarafoglou K. Matern D. Redlinger-Grosse K. Bentler K. Gaviglio A. Harding C. O. (2011). Siblings with mitochondrial acetoacetyl-CoA thiolase deficiency not identified by newborn screening. *Pediatrics* 128 e246–e250. 10.1542/peds.2010-3918 21669895

[B19] Sass J. O. (2012). Inborn errors of ketogenesis and ketone body utilization. *J. Inherit. Metab. Dis.* 35 23–28. 21479626 10.1007/s10545-011-9324-6

[B20] Sun W. Wang Y. Yang Y. Wang J. Cao Y. Luo F. (2011). The screening of inborn errors of metabolism in sick chinese infants by tandem mass spectrometry and gas chromatography/mass spectrometry. *Clin. Chim. Acta* 412 1270–1274. 10.1016/j.cca.2011.03.028 21463612

[B21] Thummler S. Dupont D. Acquaviva C. Fukao T. de Ricaud D. (2010). Different clinical presentation in siblings with mitochondrial acetoacetyl-CoA thiolase deficiency and identification of two novel mutations. *Tohoku J. Exp. Med.* 220 27–31. 20046049 10.1620/tjem.220.27

[B22] Yang C. J. Wei N. Li M. Xie K. Li J. Q. Huang C. G. (2018). Diagnosis and therapeutic monitoring of inborn errors of metabolism in 100,077 newborns from Jining city in China. *BMC Pediatr.* 18:110. 10.1186/s12887-018-1090-2 29534692 PMC5850921

[B23] Yang Y. Wang L. Wang B. Liu S. Yu B. Wang T. (2019). Application of next-generation sequencing following tandem mass spectrometry to expand newborn screening for inborn errors of metabolism: a multicenter study. *Front. Genet.* 10:86. 10.3389/fgene.2019.00086 30838026 PMC6382741

[B24] Zhang G. X. Fukao T. Rolland M. O. Zabot M. T. Renom G. Touma E. (2004). Mitochondrial acetoacetyl-CoA thiolase (T2) deficiency: T2-deficient patients with “mild” mutation(s) were previously misinterpreted as normal by the coupled assay with tiglyl-CoA. *Pediatr. Res.* 56 60–64. 10.1203/01.pdr.0000129657.48122.52 15128923

